# Extraordinarily long-inactive solitary fibrous tumor transformed to produce big insulin-like growth factor-2, leading to hypoglycemia and rapid liposarcoma growth: a case report

**DOI:** 10.1186/s12902-020-00624-2

**Published:** 2020-09-29

**Authors:** Keizo Kaneko, Shojiro Sawada, Chihiro Satake, Keiichi Kondo, Tomohito Izumi, Mamiko Tanaka, Junta Imai, Tetsuya Yamada, Hiroki Katsushima, Fumiyoshi Fujishima, Hideki Katagiri

**Affiliations:** 1 Department of Diabetes and Metabolism, Tohoku University Hospital, 2-1 Seiryo, Aoba-ku, Sendai, Miyagi 980-8575 Japan; 2 Division of Hematopathology, Tohoku University Hospital, Sendai, Aoba-ku Japan

**Keywords:** Solitary fibrous tumor, Insulin-like growth factor-2, Hypoglycemia, Tumor growth, Case report

## Abstract

**Background:**

A high-molecular-weight form of insulin-like growth factor-2 (IGF-2), known as “big” IGF-2, is occasionally produced by various tumor types, leading to hypoglycemia. Although solitary fibrous tumor (SFT) is a rare mesenchymal neoplasm, it has been estimated that 4–6% of SFT patients develop hypoglycemia due to circulating big IGF-2. The mean time elapsed from tumor detection until the onset of hypoglycemia is reportedly less than one year (8.5 ± 1.9 months).

**Case presentation:**

A 68-year-old man was hospitalized for exacerbation of recurring hypoglycemic episodes. He had been diagnosed with an SFT 17 years before the onset of hypoglycemia, and the SFT had already been very large at that time. The tumor, which was non-resectable and refractory to chemotherapies, had slowly increased in size since the initial diagnosis. Half a year before the hypoglycemic episodes manifested, another tumor, adjacent to the left kidney, was newly identified. Fluorodeoxyglucose positron emission tomography-computed tomography scanning, revealed the left peri-renal tumor to show much higher fluorodeoxyglucose uptake than the preexisting SFT, suggesting that it was unlikely to be a metastasis from the SFT. Abundant serum big IGF-2 was detected by western immunoblot analysis, indicating it to be the cause of the hypoglycemia. Since the 17 years between SFT detection and the onset of IGF-2-induced hypoglycemia was an extremely long period as compared with those in previous reports, we initially suspected that the new, peri-renal tumor had produced big IGF-2, but transcatheter arterial embolization of its feeding arteries did not suppress hypoglycemia. Notably, by measuring the tumor volume doubling time, the peri-renal tumor growth was shown to be markedly accelerated in parallel with exacerbation of the hypoglycemia. The patient died of heart failure 21 months after the onset of hypoglycemia. Unexpectedly, autopsy revealed that big IGF-2 had been produced only by the preexisting SFT, not the peri-renal tumor, and that the peri-renal tumor was a dedifferentiated liposarcoma.

**Conclusions:**

We should keep in mind that even a long-inactive SFT can undergo transformation to produce big IGF-2, which then acts on both insulin and IGF-1 receptors, possibly leading to both hypoglycemia and the development/growth of another tumor, respectively.

## Background

A high-molecular-weight form of insulin-like growth factor-2 (IGF-2), known as “big” IGF-2, is occasionally produced in various tumor types regardless of whether the origin is mesenchymal or epithelial, and can lead to hypoglycemia through insulin receptor (IR) signaling [1, 2].

Solitary fibrous tumor (SFT) is a rare mesenchymal neoplasm, the natural course of which tends to be indolent. It has been estimated that 4–6% of SFT patients develop hypoglycemia due to circulating big IGF-2 [3], and the mean time elapsed from tumor detection until the onset of big IGF-2-induced hypoglycemia is reportedly 8.5 ± 1.9 months [1]. The single most effective therapy for the hypoglycemia is a complete surgical resection of the SFT. However, this is not feasible in cases whose tumors are already massive and invasive and/or show metastasis at diagnosis.

The IGF/type1 IGF receptor (IGF-1R) signaling system is reportedly associated with tumor cell proliferation, survival and metastasis [4]. There is, however, a paucity of literature demonstrating whether big IGF-2 exerts the tumor growth effect in an endocrine manner.

We encountered a case with SFT, who, 17 years after detection of the SFT, experienced both recurring episodes of hypoglycemia and the development/growth of another novel tumor, with high circulating big IGF-2 levels.

## Case presentation

A 68-year-old man was hospitalized for exacerbation of recurring hypoglycemic episodes 18 months after the onset of these episode. He had been diagnosed with an abdominal SFT 17 years before the onset of hypoglycemia, and the SFT had already been very large at that time. The tumor was non-resectable, refractory to chemotherapies and had slowly increased in size, reaching volume of 2750 cm^3^, as measured using ITK-SNAP software [5], 8 years after the initial diagnosis.

**Fig. 1:**
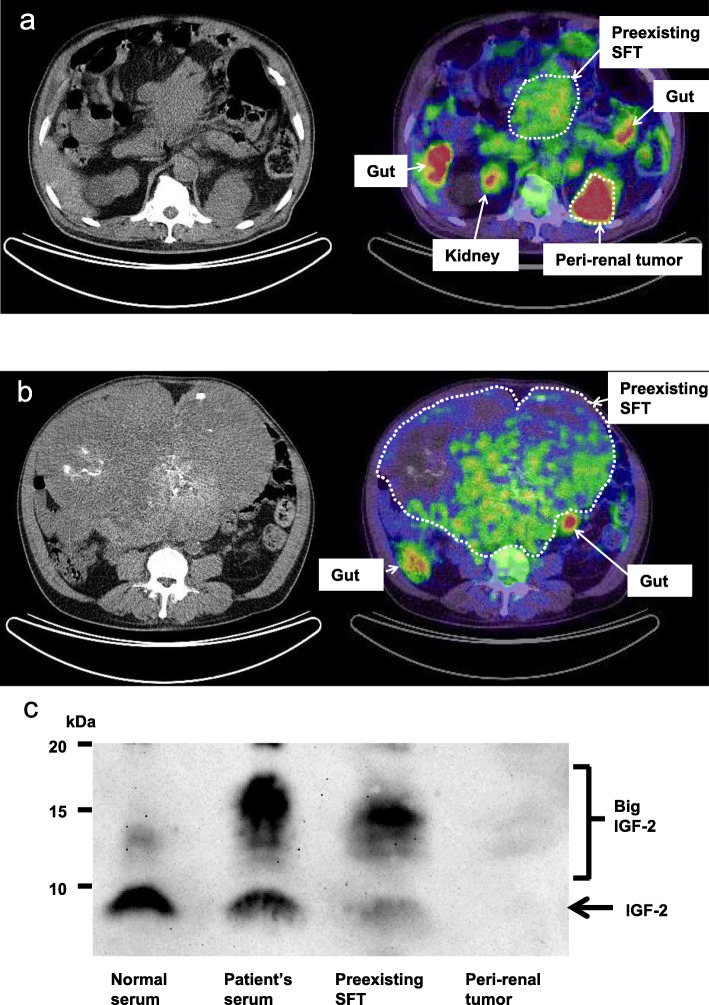
^18^F-FDG PET-CT scans of the tumors (**a**-**b**) and western immunoblot analysis with anti-IGF-2 antibody (**c**). ^18^F-FDG PET-CT scans obtained at the Th12 vertebral level (**a**) and at the L3 vertebral level (**b**) 15 months after the onset of the hypoglycemic episodes. Western immunoblot analysis with anti-IGF-2 antibody in the serum of a healthy subject (**c**, 1st lane), in the patient’s serum 18 months after the onset of hypoglycemic episodes (**c**, 2nd lane) and in the autopsy tumor samples (30 μg of total protein) of the preexisting SFT (**c**, 3rd lane) and the peri-renal tumor (**c**, 4th lane). The uncropped immunoblot image and details of the procedure including the information on anti-IGF-2 antibody are presented in Additional files 1 and 2, respectively. FDG: Fluorodeoxyglucose, PET-CT: positron emission tomography-computed tomography, IGF: insulin-like growth factor, SFT: solitary fibrous tumor

Half a year before the hypoglycemic episodes manifested, another tumor, adjacent to the left kidney, had been newly identified. Fluorodeoxyglucose (^18^F-FDG) positron emission tomography-computed tomography (PET-CT) scanning, performed 15 months after the onset of the hypoglycemic episodes, revealed much higher ^18^F-FDG uptake (standardized uptake value (SUV) max: 29) in the left peri-renal tumor than in the original tumor (SUVmax: 6.6) (Fig. 1a-b). Therefore, we considered that the peri-renal tumor was unlikely to be a metastasis from the SFT, although approximately 26 and 45% of SFT cases reportedly develop metastasis during 5- and 10-year follow-up periods, respectively [6].

**Fig. 2:**
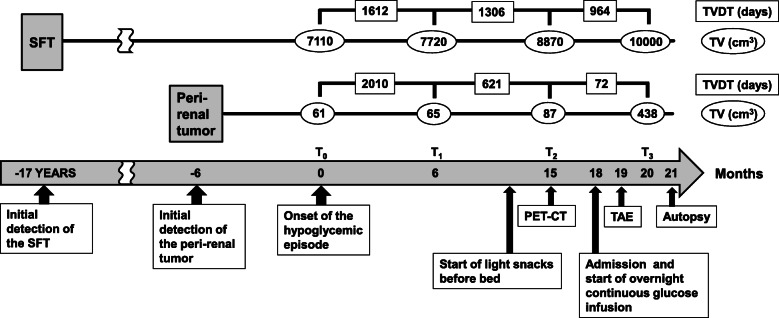
Timeline of the patient’s clinical course, including the development/growth of the tumors and hypoglycemia treatments. Tumor volumes (cm^3^) on CT scans were measured using ITK-SNAP software at four time points. TVDTs (days) were calculated as follows: TVDT = (T_y_ – T_x_) × log 2 / (log V_y_ – log V_x_). T_y_ – T_x_ indicates the time interval between the two measurements and V_x_ and V_y_ represent the tumor volumes at T_x_ and T_y_, respectively. T_0_: time of the onset of the hypoglycemic episode, T_1_: 6 months after the onset of the hypoglycemic episode, T_2_:15 months after the onset of the hypoglycemic episode, T_3_: 20 months after the onset of the hypoglycemic episode, TAE: transcatheter arterial embolization, PET-CT: positron emission tomography-computed tomography, TVDT: tumor volume doubling time, TV: tumor volume

His hypoglycemia generally manifested in the early morning or between meals. He took no glucose-lowering agents. The fasting plasma insulin level (0.2 μU/mL), compared to the plasma glucose concentration (48 mg/dL), was well suppressed (Table 1) and no pancreatic tumors were detected on detailed CT scans, likely excluding a diagnosis of insulinoma. As shown in Table 1, serum levels of counter regulatory hormones, such as adrenaline, cortisol and growth hormone, were not suppressed. The serum IGF-1 level was within the normal range. All serum liver enzyme levels were also within their normal ranges. During the current hospitalization, IGF-2 western immunoblot analysis of the patient’s serum showed a broad band between 11 and 18 kDa, showing a large amount of big IGF-2 (Fig. 1c). Its intensity was 15.6 times higher than that in a serum of a healthy subject (analyzed by ImageJ, NIH). These findings collectively indicated that big IGF-2 production was the cause of the hypoglycemia. Overnight continuous glucose infusion was started to prevent fasting hypoglycemia, following light snacks before bed, which had started a few months prior to hospital admission (Fig. 2). We considered the possibility that the newly-identified peri-renal tumor was responsible for the production of big IGF-2 and performed transcatheter arterial embolization of the arteries feeding the peri-renal tumor. However, the embolization exerted no preventive effects against hypoglycemia. Both the SFT and the peri-renal tumor continued to grow, reaching volumes of 10,000 cm^3^ and 438 cm^3^, respectively at 20 months after the onset of hypoglycemia (Fig. 2). The patient developed worsening lower limb edema due to enlargement of the preexisting SFT and his general condition deteriorated. Twenty-one months after the onset of the hypoglycemic episodes, he died of heart failure.

**Table 1:** Laboratory data

		Unit	Normal range
Glucose	48 (2.67)	mg/dL (mmol/L)	68–109 (3.78–6.06)
Insulin	0.2	μU/mL	0–18.7
Glucagon	123	pg/mL	71–174
Adrenaline	0.192	ng/mL	0–0.1
Noradrenaline	1.528	ng/mL	0.1–4.5
Cortisol	14.7	μg/dL	6.2–19.4
ACTH	19.8	pg/mL	4.4–48
GH	0.60	ng/mL	0–2.47
Insulin-like growth factor-1	100	ng/mL	70–229
Asparate aminotransferase	23	IU/L	8–38
Alanine aminotransferase	23	IU/L	4–43
γ-glutamyl transferase	18	IU/L	10–47

*ACTH* adrenocorticotropic hormone, *GH* growth hormone

**Fig. 3:**
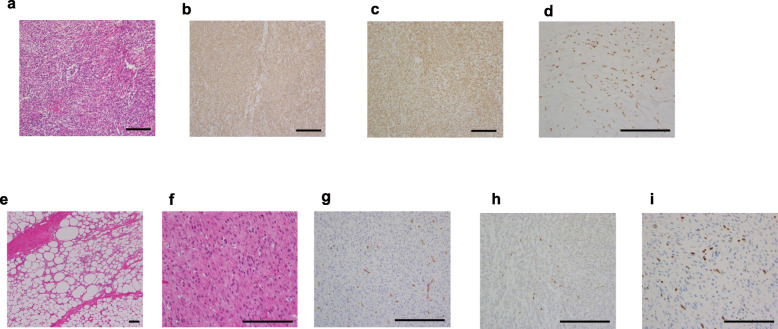
Autopsy histological findings of the preexisting SFT and the peri-renal tumor. H&E staining (**a**) and immunohistochemical staining of CD34 (**b**), bcl-2 (**c**) and STAT6 (**d**) of the preexisting SFT. H&E staining of the two components of the peri-renal tumor: well differentiated liposarcoma (**e**) and undifferentiated pleomorphic sarcoma (**f**). Immunohistochemical staining of CD34 (**g**) of the peri-renal tumor. Immunohistochemical staining of Ki67 of the SFT (**h**) and the peri-renal tumor (**i**). Bar represents 200 μm. The information on antibodies is presented in Additional file 2. SFT: solitary fibrous tumor, H&E: hematoxylin and eosin

Contrary to our expectation, western immunoblot analysis of tumor extracts obtained from his autopsy specimens showed that big IGF-2 had been produced only by the preexisting SFT, not by the peri-renal tumor (Fig. 1c). In addition, histological findings revealed the SFT to be composed of proliferative spindle cells with a component of collagen fibers (Fig. 3a). The tumor cells were immunohistochemically positive for CD34 (Fig. 3b), bcl2 (Fig. 3c) and STAT6 (Fig. 3d), typical immunohistochemical markers of SFT. On the other hand, the peri-renal tumor was morphologically composed of two components: a lesion comprised of proliferating mature adipocytes with fibrous tissues (well-differentiated liposarcoma) (Fig. 3e) and a lesion comprised of proliferative spindle cell areas with no specific differentiation, regarded as an undifferentiated pleomorphic sarcoma (Fig. 3f). Other than the endothelial cells, nearly all components were CD34-negative (Fig. 3g). Taking these observations together, we diagnosed the peri-renal tumor as a dedifferentiated liposarcoma, but not a metastasis from the SFT. Ki67-positive cell ratios of the SFT and the liposarcoma were less than 1% and approximately 15%, respectively, reflecting the rapid growth of the peri-renal liposarcoma (Fig. 3h-i).

## Discussion and conclusions

Mature IGF-2 is 7.5 kDa peptide and exerts its effects by binding to the IR or IGF-1R. In certain clinical settings, big IGF-2 (11–18 kDa) is abundantly produced by abnormal processing of an IGF-2 precursor in tumors. Big IGF-2 mainly forms a binary complex with IGF binding proteins (IGFBP), rather than the normal 150 kDa ternary complex with IGFBP-3 and an acid labile subunit, and thus can easily pass through the endothelium and gain access to the IR of parenchymal cells, leading to refractory hypoglycemia [7].

According to previous reviews, tumor types causing hypoglycemia vary from mesenchymal (e.g., leiomyoma and SFT) to epithelial (e.g., hepatocellular carcinoma) in origin [1, 2]. Among the mesenchymal tumors, the tumor type with the most prominent IGF-2 expression, in terms of both frequency and magnitude, was SFT [8]. Notably, the mean time elapsed from tumor detection until the onset of IGF-2-induced hypoglycemia is reportedly 8.5 ± 1.9 MONTHS [1]. Although the preexisting SFT was very large, the extraordinarily long period of 17 YEARS in this case prompted us to initially suspect that the peri-renal tumor was responsible for the big-IGF2 production. Contrary to our expectation, however, it was the preexisting SFT, despite its prolonged inactivity, that had produced IGF-2 and caused the patient’s recurring hypoglycemic episodes. On the other hand, the peri-renal tumor, metabolically active and rapidly growing, did not produce big IGF-2 and was found to be a dedifferentiated liposarcoma.

The IGF-1R signaling system is reportedly associated with tumor cell proliferation, survival and metastasis and has thus been extensively investigated as a target for cancer therapy [4]. In liposarcoma cases, the IGF-1R is highly expressed, and blockade of IGF signaling is reportedly one of the therapeutic targets for this disease [9, 10]. Therefore, the large amount of circulating big IGF-2 observed in our patient may have contributed to very rapid dedifferentiated liposarcoma growth with active metabolism, in addition to causing hypoglycemia. To test this hypothesis, we employed the tumor volume doubling time (TVDT) to examine alterations in the tumor growth rate after the onset of the hypoglycemic episodes (Fig. 2). TVDT, as originally described by Schwartz [11], was often calculated using tumor diameters, resulting in overestimation of the volume. The tumor volume in our case was thus measured using the ITK-SNAP software, a more accurate method based on contour segmentation of CT images [5]. Interestingly, there was a dramatical reduction in TVDT of the liposarcoma, from 2010 to 72 days, indicating a remarkably increased rate of tumor growth, associated with worsening hypoglycemia, which finally required overnight continuous glucose infusion therapy (Fig. 2). The detailed time course of the serum big IGF-2 level would further verify the importance of IGF-2 in the development/growth of the peri-renal liposarcoma.

This case exemplifies a rare but clinically significant phenomenon of biologic transformation of an SFT to secrete big IGF-2 after an extraordinary long period of dormancy, resulting in clinicopathological manifestations and possibly increased growth of another tumor.

## Supplementary information

**Additional file 1.** Uncropped western immunoblot image related to Fig. 1c.

**Additional file 2.** Procedure of protein extraction and immunoblot analysis and information of antibodies for immunoblotting and immunohistochemistry.

## Data Availability

The datasets used and/or analyzed during the current study are available from the corresponding author on reasonable request.

## Abbreviations

IGF: Insulin-like growth factor
SFT: Solitary fibrous tumor
FDG: Fluorodeoxyglucose
PET-CT: Positron emission tomography-computed tomography
IR: Insulin receptor
IGF-1R: Type1 IGF receptor
SUV: Standardized uptake value
IGFBP: IGF binding proteins
TVDT: Tumor volume doubling time
