# Supplementary material for: Extraordinarily long-inactive solitary fibrous tumor transformed to produce big insulin-like growth factor-2, leading to hypoglycemia and rapid liposarcoma growth: a case report
Source: BMC Endocr Disord. 2020 Sep 29;20:148. doi: 10.1186/s12902-020-00624-2 (PMC7526150; doi:10.1186/s12902-020-00624-2)
Supplement: Supplementary file 1 — Additional file 1. Uncropped western immunoblot image related to Fig. 1c. [file 12902_2020_624_MOESM1_ESM.pptx]

## Slide 1
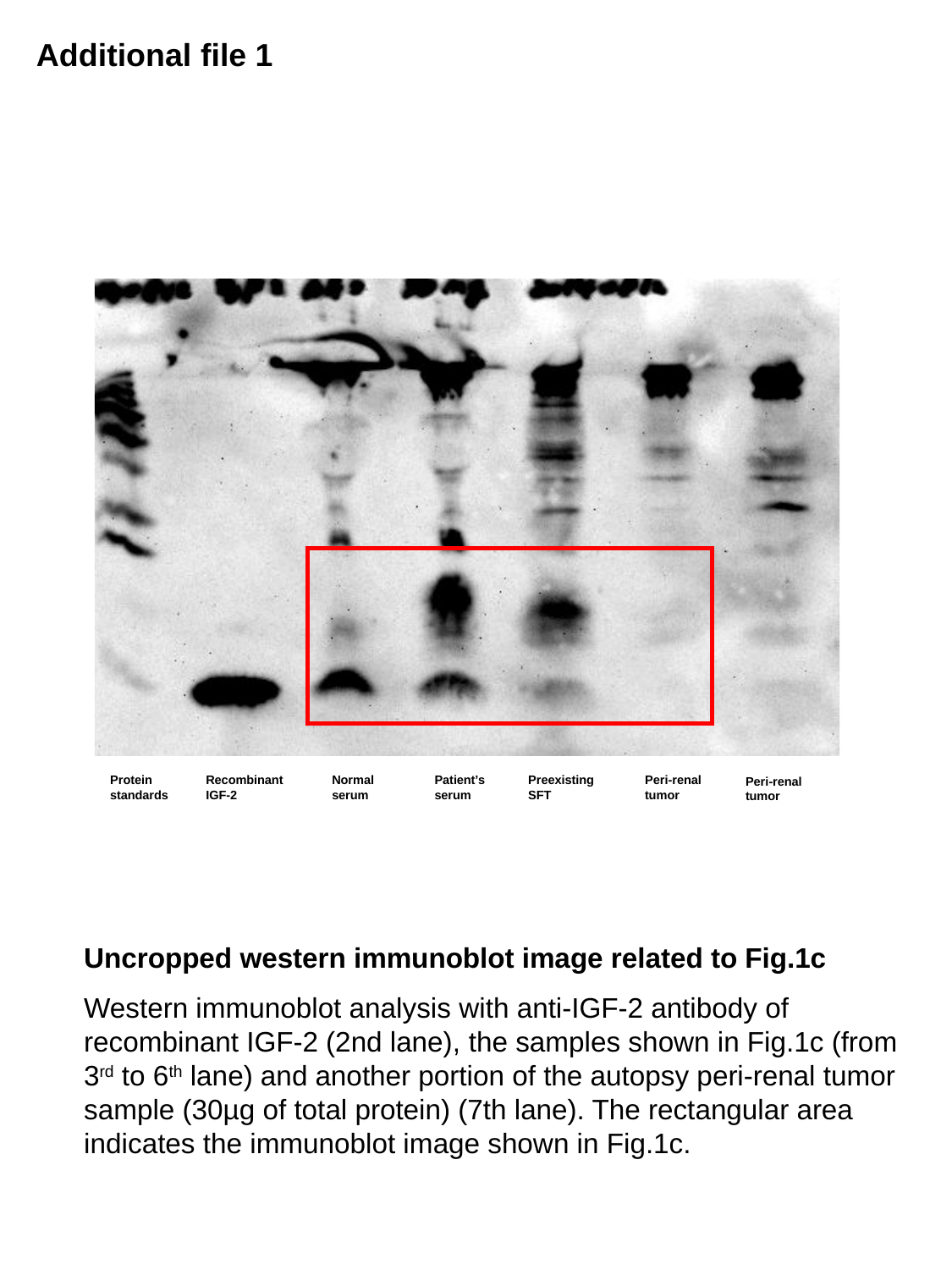

Additional file 1
Protein
standards
Recombinant
IGF-2
Normal
serum
Patient’s
serum
Preexisting
SFT
Peri-renal
tumor
Peri-renal
tumor
Uncropped western immunoblot image related to Fig.1c
Western immunoblot analysis with anti-IGF-2 antibody of recombinant IGF-2 (2nd lane), the samples shown in Fig.1c (from 3rd to 6th lane) and another portion of the autopsy peri-renal tumor sample (30µg of total protein) (7th lane). The rectangular area indicates the immunoblot image shown in Fig.1c.
