# Supplementary material for: Extraordinarily long-inactive solitary fibrous tumor transformed to produce big insulin-like growth factor-2, leading to hypoglycemia and rapid liposarcoma growth: a case report
Source: BMC Endocr Disord. 2020 Sep 29;20:148. doi: 10.1186/s12902-020-00624-2 (PMC7526150; doi:10.1186/s12902-020-00624-2)
Supplement: Supplementary file 2 — Additional file 2. Procedure of protein extraction and immunoblot analysis and information of antibodies for immunoblotting and immunohistochemistry. [file 12902_2020_624_MOESM2_ESM.docx]

**Additional file 2**

**Procedure of protein extraction and immunoblot analysis**

50 mg of tissue samples were homogenized in ice-cold lysis buffer containing 100 mM Tris, pH 8.5, 250 mM NaCl, 1% Nonidet P-40, 1 mM EDTA, 1 mM phenylmethylsulfonyl fluoride, aprotinin at 1:5,000 dilution and leupeptin at 1:5,000 dilution. Ten µL of the serum samples were diluted 10 times by ice-cold phosphate buffered saline. The Bradford method was used for the protein quantification of tumor homogenates. The supernatants of centrifuged tissue homogenates or diluted serum samples were subjected with Laemmli buffer to SDS–polyacrylamide gel electrophoresis, then immunoblotted overnight at 4°C with the IGF-2 antibody (1:2,000 dilution) followed by secondary antibodies (HRP conjugated, 1:10,000 dilution). The immunoblots were visualized with an enhanced chemiluminescence detection kit (Amersham).

The normal serum was provided by a 38-year old healthy male volunteer with normal glucose tolerance.

**Information of antibodies for immunoblotting and immunohistochemistry**

| Antibodies | Source | Identifier |
| --- | --- | --- |
| IGF-2 | Millipore | #05-166 |
| CD34 | Roche | #790-2927 |
| Bcl-2 | Roche | #790-4604 |
| STAT6 | Abcam | ab32520 |
| Ki67 | Agilent | IR626 |
